# Monosodium iodoacetate-induced monoarthritis develops differently in knee versus ankle joint in rats

**DOI:** 10.1016/j.ynpai.2019.100036

**Published:** 2019-09-03

**Authors:** Kristina Ängeby Möller, Stephanie Klein, Frank Seeliger, Anja Finn, Carina Stenfors, Camilla I. Svensson

**Affiliations:** a AstraZeneca R&D Södertälje, CNSP iMed Science, SE-151 85 Södertälje, Sweden; b Department of Physiology and Pharmacology, Karolinska Institutet, SE-171 76 Stockholm, Sweden; c AstraZeneca R&D Södertälje, Safety Pharmacology, SE-151 85 Södertälje, Sweden

**Keywords:** Arthritis, Gait analysis, Weight bearing, PawPrint, Pain, Inflammation

## Abstract

**Objective:**

Disability and movement-related pain are major symptoms of joint disease, motivating the development of methods to quantify motor behaviour in rodent joint pain models. We compared effects on behaviour, assessed the levels of biochemical mediators and made a detailed histopathological evaluation after induction of rat monoiodoacetate (MIA) monoarthritis into the ankle or knee joint.

**Design:**

Twenty-seven male Lewis rats were used. Before and up to 28 days after induction, they were tested for weight bearing during walking (dynamic), and standing (static), and for mechanical sensitivity. At termination synovial fluid was taken from ankle and/or knee joints for analysis of monocyte chemoattractant protein-1 (MCP-1), interleukin-6 (IL-6), macrophage inflammatory protein 3 alpha (MIP-3α), keratinocyte chemoattractant (KC)/human growth-regulated oncogene (GRO) and L(+)-lactate, and from separate rats joints were collected for histopathological assessment.

**Results:**

MIA ankle joint injection gave a marked reduction of dynamic weight bearing during the first days, not seen in rats with knee joint injection. At three weeks, it was decreased in the group with knee injection, but not in those with ankle injection. However, the different injection sites caused similar reductions in static weight bearing during the early phase, which was normalized in the group with ankle injection but continued and was strengthened with time in the knee injected group. Histopathological assessment, biochemical mediators and joint swelling confirmed the disparate profiles.

**Conclusions:**

This work shows that ankle versus knee joint injection of MIA resulted in different profiles in rats, which may mirror what has been found in human patients with osteoarthritis.

## Highlights

• Ankle versus knee joint injection of MIA in rats resulted in different behavioural profiles.
• Levels of biochemical mediators differs between ankle and knee injection of MIA in rats.
• Histopathological analysis show different results after ankle versus knee injection of MIA in rats.
• The rat results mirror what has been found in human patients with osteoarthritis.

## 1 Introduction

In human osteoarthritis (OA), the knee is one of the most commonly affected joints (Salaffi et al., 2014). Likewise, animal models of OA are most often focused on the knee joint, and injection of monosodium iodoacetate (MIA) into a rat knee joint, which induces chondrocyte cell death through inhibition of glyceraldehyde-3-phosphate dehydrogenase activity (Bove et al., 2003), has since the early 2000’s been described as a model of degenerative OA showing features similar to those found in patients, such as erosion and fibrillation of cartilage surface, exposure of subchondral bone, formation of osteophytes and disorganization of chondrocytes (Guingamp et al., 1997, Kobayashi et al., 2003).

The model causes pain-like behaviours and is associated with reduced spontaneous locomotor behaviour, reduced static weight bearing of the injected limb (Bove et al., 2003, Guingamp et al., 1997, Kobayashi et al., 2003, Pomonis et al., 2005), mechanical hyperalgesia and tactile allodynia (Fernihough et al., 2004). Thus, the MIA model has provided means of investigating possible mechanisms of OA pain (Ivanavicius et al., 2007, Sagar et al., 2011, Havelin et al., 2016), and has been used to assess efficacy of existing antiarthritic agents and analgesics (TenBroek et al., 2016), as well as of much needed new treatments (Nwosu et al., 2016, Comi et al., 2017, Jimbo et al., 2019). However, we have previously shown that inducing inflammation in the ankle joint using Freund’s complete adjuvant (CFA) or carrageenan resulted in more pronounced pain-like behaviour of rats in motion, compared to when the inflammation was induction in the knee joint (Ängeby Möller et al., 2012), and the aim of the present study was to investigate whether this holds true also for the MIA monoarthritis model.

In human OA patients, disability and movement related pain are major complaints (Dieppe and Lohmander, 2005, Bijlsma et al., 2011), and pain on walking has been used as an endpoint to assess treatments in clinical trials (Lee et al., 2004, Conrozier et al., 2006, Lane et al., 2010). Despite animals being four-legged and obviously different from two-legged humans in many aspects, the rodent models partly mimic the clinical situation with regard to tissue injury and show movement related pain originating from the joint, and may facilitate investigation of disease mechanisms and aid development of new treatments.

Today, it is widely accepted that inflammation plays a part in the development and symptoms of OA, and chemokines and cytokines have been suggested to be involved in the progression of OA (Goldring and Otero, 2011). These include monocyte chemoattractant protein-1 (MCP-1), macrophage inflammatory protein 3 alpha (MIP-3α), interleukin-8 (IL-8) and IL-6 which are assessed here. The pro-inflammatory chemokine MCP-1 regulates migration and infiltration of monocytes/macrophages (Deshmane et al., 2009), and has been shown to enhance excitability in nociceptive neurons (Sun et al., 2006), while MIP-3α is strongly chemotactic for lymphocytes but also attracts neutrophils (Hieshima et al., 1997). Through the recruited cells, IL-8 and IL-6 are secreted. An increase in osteoclastic activity with consecutive bone resorption through an IL-8-dependent autocrine loop has been demonstrated (Kopesky et al., 2014), suggesting that the rodent analogue to human IL-8, keratinocyte chemoattractant (KC)/human growth-regulated oncogene (GRO) could have the same function. Once released, IL-6 stimulates bone resorption and osteoclast formation (Fonseca et al., 2009). In addition, increased levels of IL-6 has been found in OA patients (Monibi et al., 2016), and is suggested to predict for development of OA (Livshits et al., 2009, Larsson et al., 2015). Increased levels of lactate in synovial fluid, reflecting inflammatory activity (Finn and Oerther, 2010, Finn et al., 2014), have been shown in OA, as well as in septic arthritis and in experimentally induced osteonecrosis (Finn and Oerther, 2010).

The hallmarks of human OA, primarily affecting weight-bearing joints such as knees and hips (Bove et al., 2003); are progressive breakdown of articular cartilage as well as remodelling of subchondral bone (Salaffi et al., 2014, Loeser et al., 2012, Burr and Gallant, 2012). Injection of MIA into the joints gives suppression of chondrocyte metabolism by inhibition of glyceraldehyde-3-phosphate dehydrogenase activity, subsequent impairment of glycolysis and cell death (Bove et al., 2003). Associated is the loss of proteoglycan, a glycosylated protein present in the extracellular matrix of cartilage with major hydrodynamic function providing swelling pressure to the tissue enabling it to withstand compressional forces (Yanagishita, 1993). Cartilage erosion with exposure and sclerosis of subchondral bone, osteophyte formation, functional joint impairment and production of pro-inflammatory factors follow during the progression of the model (Finn et al., 2014), and the loss of chondrocytes in the articular cartilage resembles histomorphological changes seen in human OA.

The aim of this study was to compare the effects of MIA-induced monoarthritis in the ankle versus the knee joint on behavioural readouts. The inflammatory response (through assessing levels of MCP-1, MIP-3α, KC/GRO, IL-6 and L(+)-lactate in the synovial fluid), and the histopathology of the animals was determined at termination.

## 2 Materials and methods

### 2.1 Animals and housing

**Fig. 1:**
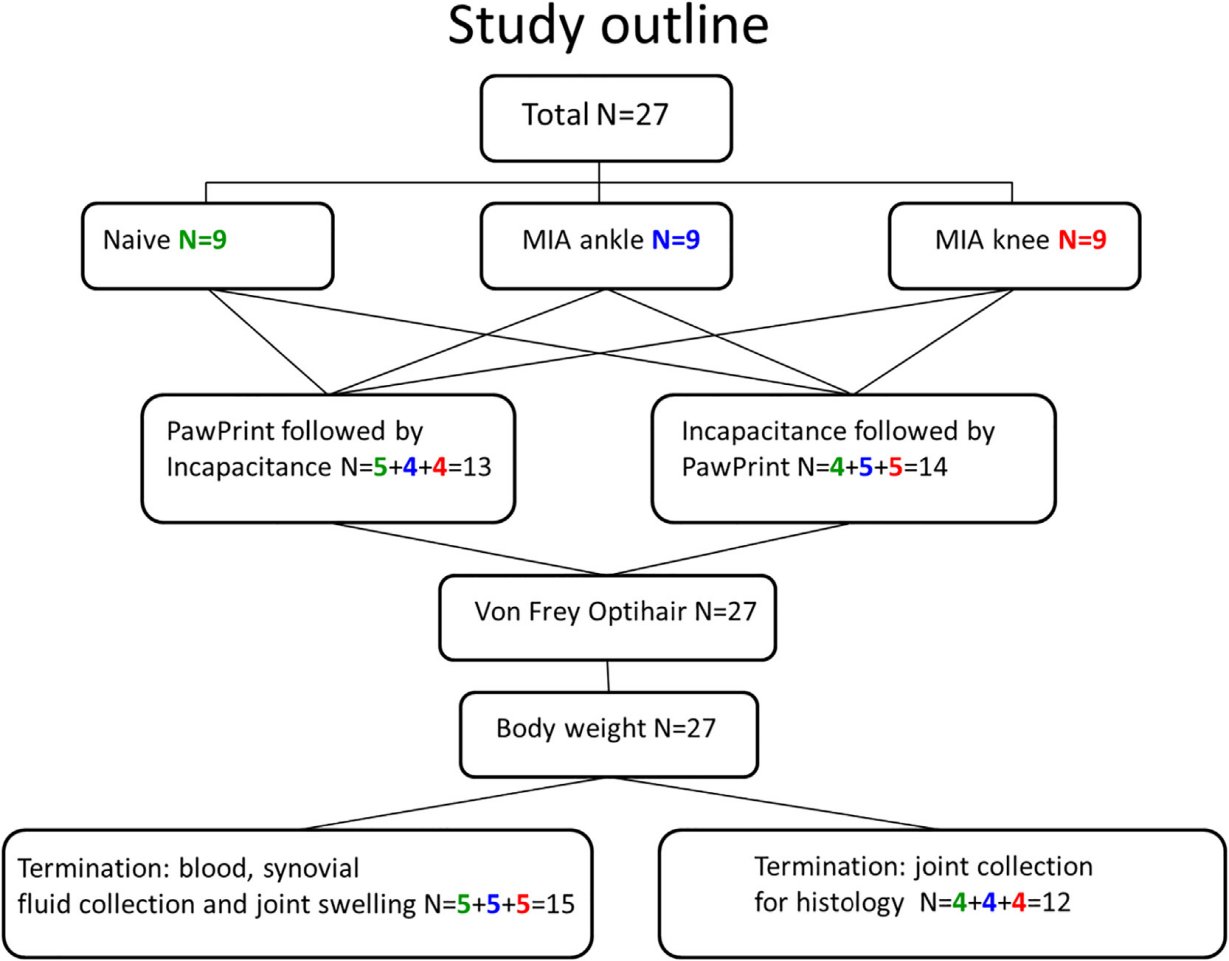
The study outline.

The outline of the study is shown in Fig. 1. In total, 27 Lewis inbred male rats, (Harlan Laboratories BV, Horst, The Netherlands), divided into three groups of nine and weighing 250–280 g at the start of testing were used. Before and after induction of monoarthritis, all rats were subjected to behavioural testing. At termination of the in-life phase synovial fluid was collected from 5 rats from each group, and joints were taken from the remaining 4 rats/group. The animals were housed 4 per cage in Macrolon® cages with wood shavings as bedding, with free access to food (R70, Lactamin AB, Vadstena, Sweden) and tap water. The lighting was controlled with 11.5 h daylight, 11.5 h darkness, 0.5 h dusk and dawn. The animals were acclimatized for at least one week before being subjected to experimental procedures. Handling and testing were performed during the light phase in a room with dimmed lights. Treatments were randomized by a computer program and the observer was blinded to group assignment, however due to swelling caused by the MIA injection it cannot be excluded that this was not a complete blinding. These studies were approved by the Stockholm Södra Animal Research Ethical Board. All animal experiments comply with the ARRIVE guidelines and were carried out in accordance with the EU Directive 2010/63/EU for animal experiments. All efforts were made to minimise animal suffering and to reduce the number of animals used. No alternatives to the in vivo techniques used here were available.

### 2.2 Induction of monoarthritis

Under deep anaesthesia (5% isoflurane in oxygen/breathing air), 50 µL physiological saline containing 2 mg MIA (Monosodium Iodo-acetate, Sigma-Aldrich) was injected with a 21-gauge needle into the left tibio-tarsal joint or into the left knee joint.

### 2.3 Handling and behavioural testing procedures

The animals were habituated in the test room for at least 30 min before testing. One day before induction baseline tests were performed. Testing was done in a cross-over design where 4–5 rats from each treatment group were first assessed on the PawPrint setup and then on the Incapacitance tester, whereas the remaining rats from each treatment group were assessed in the reverse order. Subsequently all rats were tested for mechanical sensitivity.

*Assessment of dynamic weight bearing:* Training on the test equipment was performed six and three days prior to monoarthritis induction. Testing was performed as described in detail previously (Ängeby Möller et al., 2012), in a custom setup built at AstraZeneca, Södertälje, similar to the CatWalk system but which included an automated analysis. In short, rats were allowed to cross a passage (length 100 cm, width 10 cm, height 21 cm) with a glass floor where light is projected into the long edges. When a rat paw touches the glass, an illuminated image is produced. The light intensity of the image reflects the pressure exerted. Data acquisition and analysis based on video capture was done using the PawPrint algorithm from which dynamic weight bearing of the MIA injected paw was obtained in the following way: Pixels showing intensities above a threshold value of 80 (range 0–255, arbitrary unit) were defined as contact points and were automatically assigned to the relevant paw. For the duration of each paw placement, the maximum value of light intensity was recorded for each pixel. The sum of these values was considered to be the dynamic weight bearing of the particular paw placement. For each paw, the median of all steps captured in a passage was calculated and the sum of all four paw’s medians was defined as the rat’s total dynamic weight bearing. The weight bearing of each paw is shown in percent of this value.

*Assessment of static weight bearing:* Habituation to the Incapacitance tester (Linton Instruments, Palgrave, UK) was done for 5 min once before the testing started. The animals were placed in restrainers with their hind paws on separate sensors registering the weight of each hind paw, and allowed to settle for about one minute before 5 recordings, each lasting 3 s, were made. The mean value of the 5 recordings were used, and data for each hind paw was expressed in percent of both hind paws’ static weight bearing.

*Assessment of mechanical sensitivity:* von Frey filaments (OptiHair, MARSTOCK nervtest, Schriesheim, Germany) in a series of eight filaments with logarithmically incremental stiffness (2.8, 4.0, 5.7, 8.0, 11.3, 16.0, 22.6, 32.0 g) were used. Rats were placed on a wire mesh grid which allowed access to the paws, and were left to habituate for 15–30 min. The filaments were applied to the mid-plantar left hind paw from underneath the grid floor perpendicular to the plantar surface until slight buckling occurred, and held for 4–6 s. A positive response was noted if the paw was withdrawn. Testing was started with the filament possessing a buckling force of 8.0 g, and the 50% withdrawal threshold was determined using the up-down method (Dixon, 1980, Chaplan et al., 1994).

### 2.4 Effect on general wellbeing and assessment of inflammation

To assess general health of the animals, body weight was measured before and 11, 19, 22, 25 and 28 days after injection of MIA.

At termination of the study, the animals were sacrificed by intracardiac injection of pentobarbital. The diameter of ankle or knee respectively was assessed, taking the mean of 3 measurements, using a three-button digital calliper (Limit, Alingsås, Sweden).

### 2.5 Biochemical analysis

At termination, the first five animals per treatment were used to collect synovial fluid from the injected joint. The skin above the ankle or knee joint was opened transversally with a scalpel, the ligament above the joint sectioned and the cavity rinsed 4 times with 25 μL 0.05 M EDTA, pH 7.5. The fluid was centrifuged at 4 °C, (3000 rpm; 10 min), and the supernatant analysed for biochemical mediators.

Levels of MCP-1, MIP-3α, KC/GRO, and IL-6 were analysed using custom-made immunoassay kits (Meso Scale Discovery, Rockville, MD, USA), tested and validated following the manufacturer’s instructions. All samples were randomized before assay procedures. Synovial fluid samples were diluted 1:4 in assay diluents before adding them to the plates, and analyzed according to the manufacturer’s protocol. The lower limits of quantification (LOQ), corrected for dilutions, were 624 pg/ml for MCP-1, 39 pg/ml for MIP-3α and KC/GRO, and 79 pg/ml for IL-6.

L(+)-lactate levels were measured by a colorimetric assay (K607, BioVision, Milpitas, CA, USA) described previously (Finn et al., 2014). In brief, samples diluted up to 1:32 with kit buffer were transferred to a 96-well microplate and mixed with an equal volume of the provided reaction mix. After 30 min of incubation at room temperature, the microplate was read at 570 nm on a Spectramax 340 (Molecular Devices, Sunnyvale, CA, USA). The LOQ was 0.02 mmol/l.

### 2.6 Histopathological assessment

From the remaining four animals per treatment, both hind leg joints (ankle or knee; treated and untreated) were taken immediately after euthanasia. Skin and soft tissues were carefully removed and the diaphyses carefully opened at least 1 cm proximal and distal of the ankle or knee joint to ensure rapid internal fixation. The prepared joints were fixed in 4% phosphate buffered paraformaldehyde (Histolab Products AB, Gothenburg, Sweden) for at least 24 h. For decalcification of the mineralized tissue, the joints were transferred into EDTA (ethylenediaminetetraacetate 0.1 mol/l, pH 7.4; Sigma-Aldrich Sweden AB, prod. no. 34550) for at least 2 weeks. Ankle and knee joints were longitudinal trimmed for representative overview of weight bearing cartilage. The cut of the tibio-tarsal joint was performed between the metatarsals II and III as previously described (Bolon et al., 2011), while the knee joint was cut longitudinal at the level of the lateral femur condylus. The tissues were then embedded in paraffin and cut in 4 µm serial sections.

Detection of degenerative-inflammatory changes in MIA-injected ankle and knee joints of all animals was assessed based on H.E. stained slides and compared with naïve joints. A standardized Safranin-O stain was used to detect loss of proteoglycan in the cartilage extracellular matrix on ankle and knee joints of treated and naïve animals. All stained-glass slides were scanned with a Hamamatsu scanner (Hamamatsu NanoZoomer Digital Pathology-NDP) using x20 magnification. Light microscopic qualitative analysis was performed using a Zeiss Axioplan 2 microscope, and representative pictures were taken using Aperio Image Scope v11.2.0.780.

### 2.7 Data analysis and statistics

All statistical analyses were performed using GraphPad Prism 6.03. Data are presented as mean values ± 95% confidence interval (CI; n = 9 per group unless otherwise stated), except for results from analysis of biochemical mediators which are presented as individual values and medians. The results obtained from the behavioural tests and body weight changes were subjected to 2-way repeated measures analysis of variance (ANOVA), followed by Bonferroni’s multiple comparison test. Joint swelling results were analysed using unpaired *t*-test. Levels of L(+)-Lactate was subjected to Kruskal-Wallis test, with subsequent post hoc comparisons using Mann-Whitney test. A *p* value of less than 0.05 was considered significant.

## 3 Results

### 3.1 Nociceptive behaviour

#### 3.1.1 Dynamic (PawPrint) and static (Incapacitance tester) weight bearing after MIA injection in ankle and knee

**Fig. 2:**
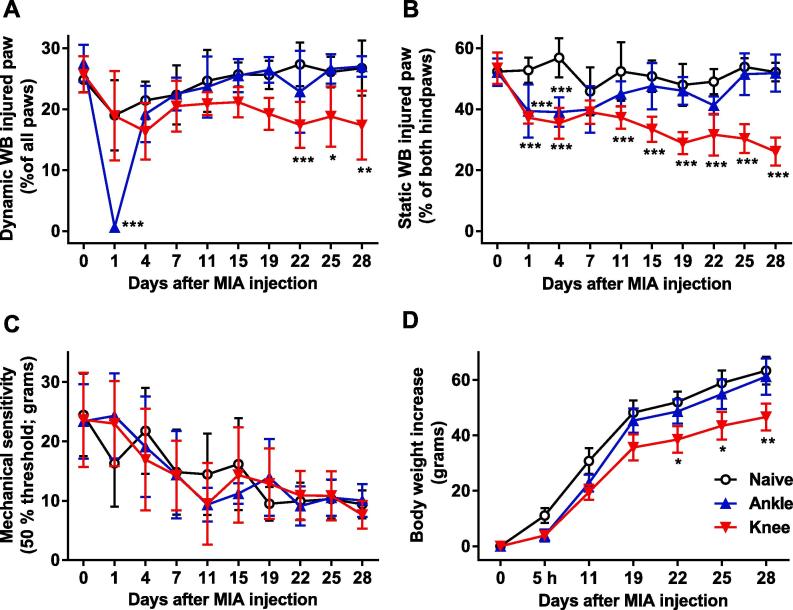
Time course of (A) the dynamic weight bearing, (B) the static weight bearing, (C) the mechanical sensitivity of the injected left hind paw, and (D) the body weight development in naïve control rats and rats before and after induction of monoarthritis by injecting MIA into the ankle or knee joint. Bonferroni’s test subsequent to 2-way RM ANOVA: * = *p* < 0.05, ** = *p* < 0.01, *** = *p* < 0.001. Data shown as mean ± 95% CI, n = 9 per group.

In naïve rats, neither the dynamic (while walking) nor the static (at standing) weight bearing of the left hind paw changed during the course of the study compared to baseline values (Fig. 2A, B).

A significant difference in the dynamic weight bearing of the injected paw in the PawPrint assay was found between the treatment groups (Fig. 2A; p < 0.001 for group and time effects and interaction). In the group with ankle injection, a marked reduction of mean dynamic weight bearing from 27.6% (24.6–30.6; 95% CI) before, to 0.6% (0.4–1.3; 95% CI) one day after the injection was observed, after which it was normalized and remained at the same level as the naïve group until termination of the study. In contrast, injection of MIA into the knee led to a slowly progressing change in dynamic weight bearing of the injected paw that was observed from 22 days after injection and lasted until termination. Though longer lasting, the effect of knee injection on dynamic weight bearing was less pronounced, reaching 17–18% of all four paw’s total weight bearing (Fig. 2A).

Static weight bearing was also significantly altered by injection of MIA (p < 0.001 for group and time effects and interaction). Injection of MIA to the ankle and knee joint induced similar levels of reduction in weight bearing one and four days after injection (from 50% down to 37–39% of the injected hindpaws’ weight bearing). While the static weight bearing after injection into the ankle joint was normalized by day 7, MIA injected into the knee joint led to a reduction which was statistically significant from day 11 through day 28 (Fig. 2B).

#### 3.1.2 Mechanical sensitivity

Injection of MIA did not induce mechanical hypersensitivity (p = 0.9823 for group effect, p < 0.001 for time effect and p = 0.5588 for interaction). All groups, however, showed increased sensitivity over time, with a progressing reduction in the 50% withdrawal threshold from about 25 g on the first test day to 10 g day 28 (Fig. 2C).

### 3.2 Body weight development

The body weight of naïve control rats increased with about 60 g during the four-week study. Both MIA and injection site affected weight gain (p = 0.0045 for group effect, p < 0.001 for time effect and interaction). Those receiving ankle joint MIA injection gained weight at the same rate as naïve rats, whereas animals with knee joint MIA injection showed significant less body weight gain from 22 days onwards (Fig. 2D).

### 3.3 Joint swelling

**Fig. 3:**
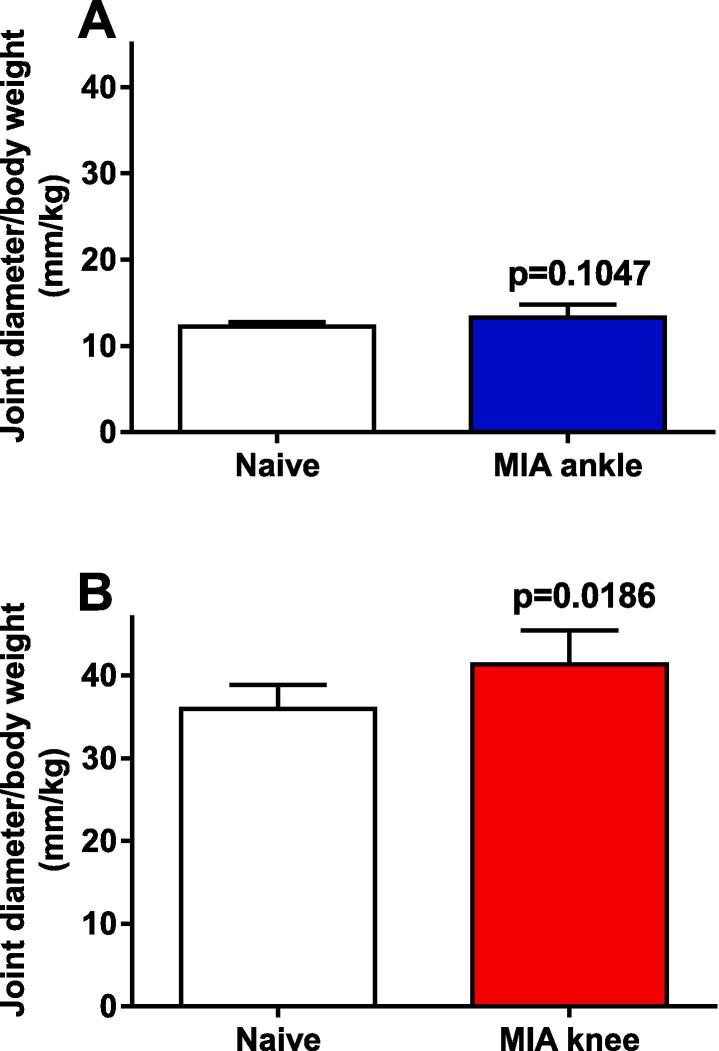
Diameter of ankle (A) and knee (B) joints measured in rats 28 days after injection of MIA. Both ankle and knee joints were measured in the naïve control rats. Unpaired *t*-test was performed and subsequent p-values shown. Data shown as mean ± 95% CI, n = 5 per group.

Swelling was assessed at termination as joint diameter adjusted for individual body weight. No increase of the ankle-injected joint diameter was observed (Fig. 3A; p = 0.1047), but an increase in joint diameter of the knee joint injected with MIA was detected compared to non-injected joint or to naïve animals (Fig. 3B; p = 0.0186).

### 3.4 Biochemical mediators

**Fig. 4:**
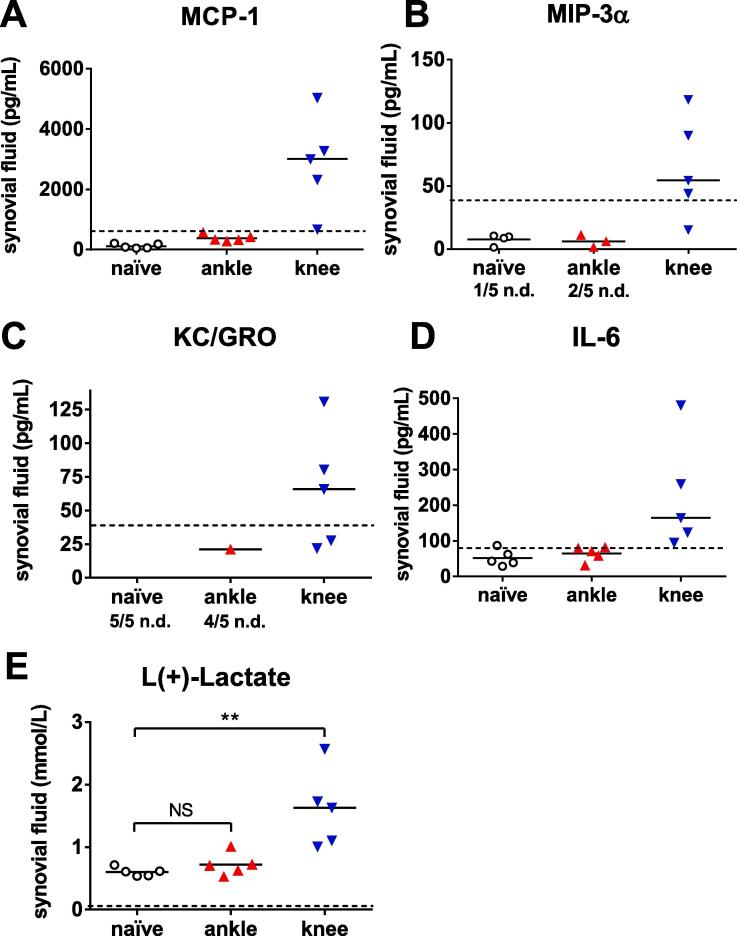
MCP-1 (A), MIP-3α (B), KC/GRO (C), IL-6 (D) and L(+)-lactate (E) assessed in synovial fluid collected from naïve control rats and from rats 28 days after injection of MIA into the ankle or knee joint. MIP-3α (B) could not be detected (stated as n.d. in the figure) in 1/5 of the naïve rats, nor in 2/5 in the rats injected into the ankle joint. KC/GRO (C) was not possible to detect in any of the 5 naïve rats or in 4/5 rats with ankle joint injection. Mann-Whitney test subsequent to Kruskal-Wallis test performed for L(+)-lactate: ** = *p* < 0.01. Data shown as individual values and median, n = 5 per group. n.d. = not detectable.

In synovial fluid from naïve rats, levels of MCP-1, MIP-3α, KC/GRO or IL-6 were below LOQ, while the median level of L(+)-lactate was 0.603 mmol/l. Injection with MIA into the ankle joint did not result in an increase in L(+)-lactate nor quantifiable levels of MCP-1, MIP-3α, IL-6 or KC/GRO in synovial fluid 28 days after injection. In contrast, synovial fluid from rats injected with MIA into the knee joint had increased levels of all biochemical mediators, statistically significant for L(+)-lactate (Fig. 4).

### 3.5 Histopathological evaluation

**Fig. 5:**
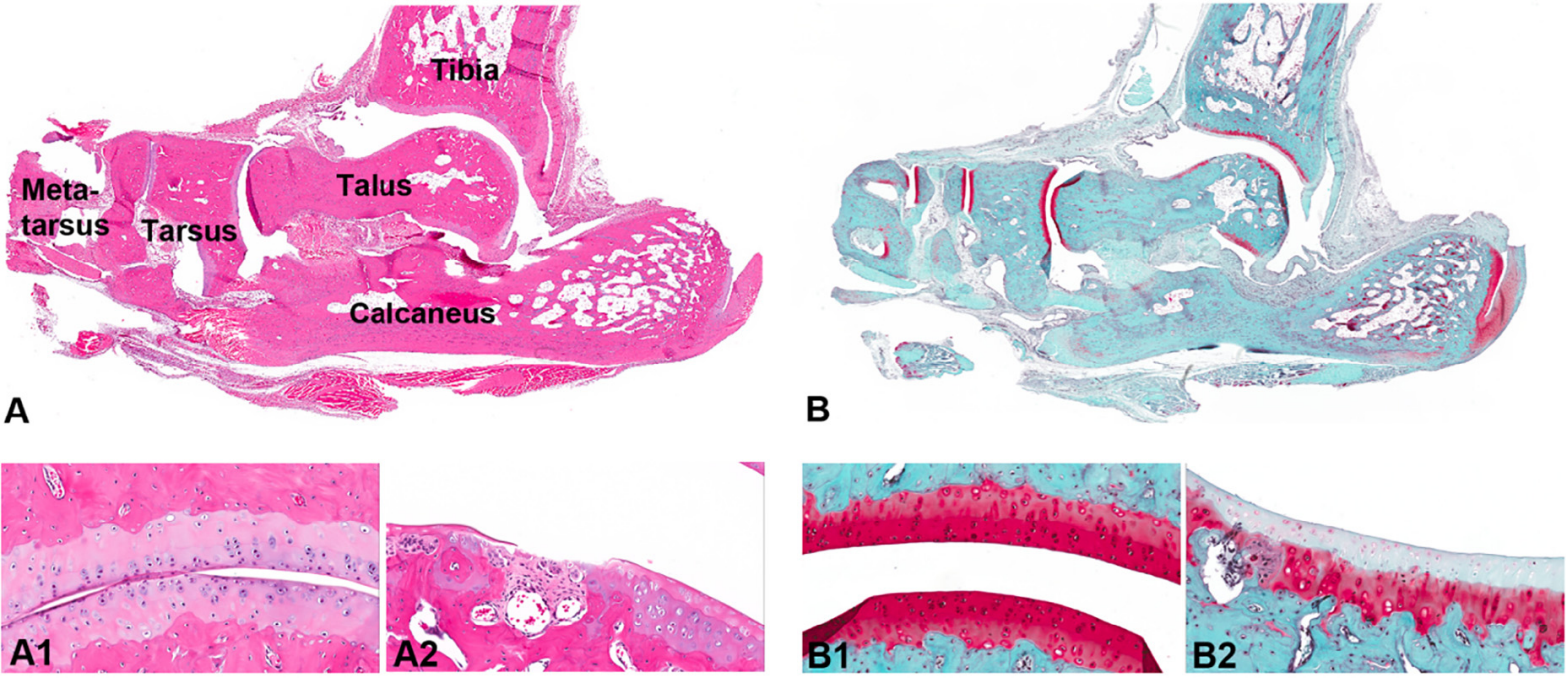
HE staining (A) and Safranin-O staining (B) show minimal histopathological lesions observed in the ankle joint after MIA injection. Healthy hyaline cartilage covering the talotarsal surface containing intact chondrocytes embedded in healthy ground substance is shown in A1 (HE staining), and confirmed by Safranin-O staining in B1. Figures A2 and B2 show focal articular ulceration seen on the talar surface. Loss of viability in superficial chondrocytes is accompanied by loss of superficial proteoglycan (HE staining and Safranin-O staining, respectively).

Twenty-eight days after MIA ankle injection, very discrete, degenerative lesions were found in the tibio-talar joint (Fig. 5). Moderate loss of proteoglycan confirmed by Safranin-O staining was a common finding. Only one of four animals displayed mild focal erosion at the talar surface (Fig. 5A.2 and B.2). The talo-navicular joint showed no lesions, except in one animal where minimal to mild loss of proteoglycan was detected.

**Fig. 6:**
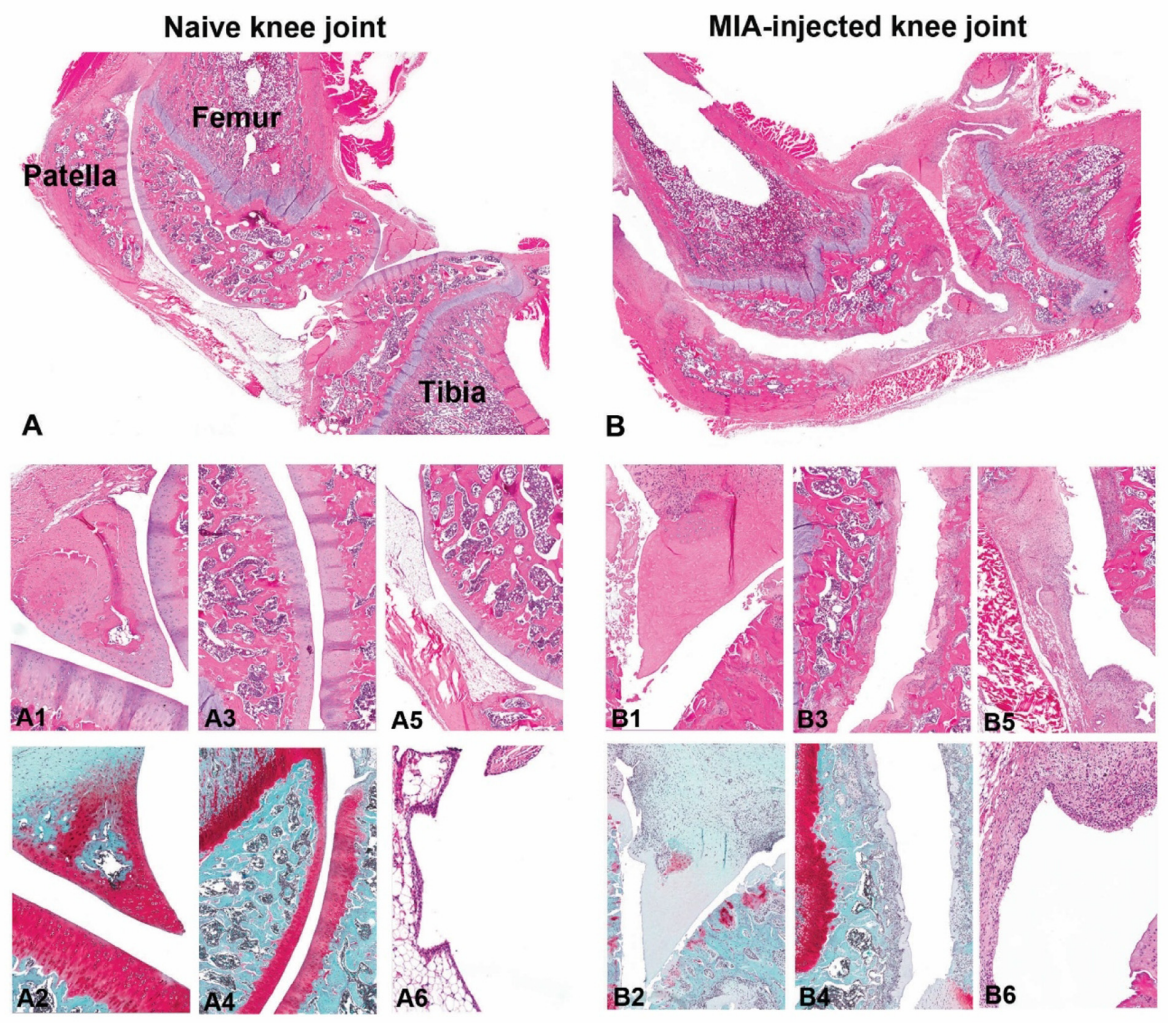
Overview of a healthy, naïve knee joint (A), in comparison to a MIA-injected, osteoarthritic knee joint (B). Figures A1 and A2 show healthy chondrocytes forming a triangular meniscus (A1: HE staining; A2: Safranin-O staining). Necrotic chondrocytes and loss of shape was observed in the meniscus after MIA injection (B1: HE staining) accompanied with loss of proteoglycan shown in Safranin-O staining (B2). The femoropatellar articulate surface in naïve joint is shown in figures A3 (HE staining) and A4 (Safranin-O-staining). Degenerative, diffuse, chronic erosive changes were seen in MIA-injected joints (B3: HE staining), confirmed with Safranin-O staining in B4. A5 (HE staining) shows the healthy subpatellar fat pad in comparison to the replacement of the fad pad with connective tissue overlined by synovial hypertrophy in MIA-injected joints (B5). Normal synovial epithelium in naïve rat is shown in A6, compared to the synovial hypertrophy and sub-synovial granulation tissue accumulation observed in the MIA-injected knee joint (B6).

After MIA knee injection severe diffuse erosive-ulcerative changes were found in the femoro-patellar and in the femoro-tibial joints of all four animals (Fig. 6B) compared to the control rats (Fig. 6A). The articular cartilage was eroded in weight bearing areas, and the few remaining cartilage rims showed multifocal fibrillation. A ghost-cell-like morphology was found for the remaining chondrocytes, and an overall loss of proteoglycan in the cartilage matrix was confirmed by Safranin-O stain. Osteophytes developed, and subchondral bone structures of femur, tibia and patellar displayed erosive lesions. Moreover breakdown of trabecular bony structures was obvious in all four rats, with occurrence of very few subchondral cysts in severe lesions. Reactive changes were characterized by increased amounts of fibroblasts placed within connective tissue, and appearance of large, multinucleated osteoclasts attached at pre-existing trabecular bone next to areas with activated osteoblasts encircled by newly-produced osteoid. The epiphyses, patella and menisci were strongly deformed, and the synovial epithelial cells showed moderate hypertrophy, while the infra-patellar fat tissue was displaced by moderate connective tissue.

## 4 Discussion

The aim of this study was to compare effects on behavioural readouts, some inflammatory mediators and joint pathology after injection of MIA into the ankle joint or the knee joint. MIA-induced monoarthritis in the ankle joint led to a pronounced but transient behavioural response lasting a few days for both dynamic and static weight bearing. In contrast, injection of MIA into the knee joint led to a weaker immediate behavioural impairment that increased with time and was still present at day 28. In rats injected into the knee joint, a statistically significant response was seen earlier in the static weight bearing than in the dynamic weight bearing. Previous reports showed that MIA injection into the knee joint resulted in a biphasic response, with an early inflammatory reaction followed by damage of the entire cartilage seen two weeks later (Bove et al., 2003). Accordingly, we found a tendency of a biphasic course in the static weight bearing assay in animals injected into the knee. In contrast, only one phase of pain-like behaviour was observed after MIA-injection to the ankle joint, suggesting that development of joint pathology in rats is joint dependent.

To our surprise injection of MIA to the ankle joint resulted in a transient, but pronounced weight bearing reduction, whereas injection into the knee joint gave a modest reduction detectable only after three weeks. This implies that the ankle joint is more susceptible to inflammation in the first phase, much like results shown for monoarthritis induced by the two inflammatory agents CFA and carrageenan (Ängeby Möller et al., 2012). In addition, there were differences in the effects on static compared to dynamic weight bearing. The static weight bearing was instantly impaired in both groups, but resolved completely in rats injected into the ankle joint. The rats with knee MIA-injection showed reduced static weight bearing at day one and 4, which was resolved on day 7 but which was then slightly but continuously reduced over time. This may reflect that a shift from inflammation towards injuries in the cartilage and underlying bony structures occurred in the knee, possibly leading to exposure of the sensory nerve endings in the subchondral bony tissue.

Weight bearing pain as compared to non-weight bearing pain has been strongly correlated with denuded cartilage area over subchondral bone in the knee of human patients (Cotofana et al., 2013), and a regrowth of sensory nerve fibres along with sprouting of new blood vessels during the osteochondral remodelling in OA progression has been demonstrated in rats (Suri and Walsh, 2012). It can be argued that the weight an animal chooses to put on a hind paw with an injured or inflamed joint is affected by different stimuli; one being the load itself exerting pressure on the joint tissues, and the other consisting of the friction as different surfaces inside the joint meet in movement. In the situation of assessing static weight bearing the animal stands on both legs without moving, and the painful stimuli thus most likely is that of the load on the joint. Under these circumstances, no major difference in effects of MIA induced ankle or knee inflammation during the first phase would be expected. In contrast, when the rat is walking, the joint surfaces need to rub against each other. Thus, in moving rats both stimuli could add to the nociceptive signal and the site of inflammation would make a difference. The ankle joint is close to the floor and needs to be bent at every step, and there are no strategies available to reduce the joint friction unless the paw is partially or completely lifted. When the knee joint is affected, there are ways to avoid the friction by placing the paw at a larger distance from the body and using other joints such as the hip and/or ankle. Using more detailed measures of gait parameters and kinematics, which was not possible with the PawPrint setup, Lakes and Allen (Lakes and Allen, 2018) have indeed recently shown that rats with knee joint injection of MIA have wider hind step widths, together with a decrease in stride length.

In contrast to previous reports (Fernihough et al., 2004), this study showed no effects on mechanical sensitivity after ankle or knee intra-articular MIA injection. This discrepancy could depend on the performer, as different testers have been shown to significantly affect the level of response (Sorge et al., 2014), or on stress due to the many tests the rats were exposed to each day (Butler and Finn, 2009) (dynamic and static weight bearing as well as mechanical sensitivity), but needs further evaluation to be fully understood.

The biochemical inflammatory mediators measured in the synovial fluid and the joint swelling confirmed the disparate profiles in the ankle and knee joints. No changes in concentrations compared to naïve rats were found from rats injected with MIA into the ankle joints, but levels of L(+)-lactate, and the pro-inflammatory markers MCP-1, MIP-3α, KC/GRO and IL-6, were elevated 28 days after MIA injection into the knee joint. This is in agreement with a previous study (Finn et al., 2014), where MIA induced distinct inflammatory markers in a biphasic manner, and confirms the biphasic changes in MIA injected knees as described by Bove in 2003^2^. Mononuclear cells were detected in small amounts in the synovium on day 28 post MIA knee-injection and multinuclear phagocytic cells such as osteo- and chondroclasts were found in reasonable numbers in the subchondral bone. Cytokines produced by chondrocytes, mononuclear cells, osteoblasts and synovial tissues are involved in the pathophysiology of osteoarthritis (Kapoor et al., 2011), and have direct effects on nociceptive neurons in addition to their pro-inflammatory action (Schaible, 2014). The increased release of MCP-1, KC/GRO and IL-6 found in this study may have added to the pain sensation, as those can sensitize or depolarize neurons (Sun et al., 2006, Dong et al., 2012, DeLeo et al., 1996, Obreja et al., 2002, Brenn et al., 2007).

The MIA concentration used here caused only very mild lesions in the rat ankle joint. However, when injected into the rat knee joint it led to lesions similar to those described for human OA “end-stage” patients that are qualified for a total knee arthroplasty (Zeni et al., 2010), defined as severe pain, higher levels of disability and excessive cartilage degeneration based on a Kellgren Lawrence (Kellgren and Lawrence, 1957) score ≥ 3. Thus, our results from the rat OA model showed differences between joints in a similar way as in the human OA, where the knee is more affected than the ankle (Salaffi et al., 2014, Felson, 1995, Kuettner and Cole, 2005). The ankle joint from healthy humans contains more proteoglycan and less water than the knee joint, and is less susceptible to catabolic factors (Eger et al., 2002), and it has been hypothesized that the higher rate of proteoglycan synthesis and turnover in the ankle joint leads to reduced permeability and increased stiffness (Kuettner and Cole, 2005). Ankle joint cartilage explants from healthy human donors were less sensitive to damage by fibronectin fragments and had greater capacity for repair compared to the knee joint (Salaffi et al., 2014, Kuettner and Cole, 2005, Kang et al., 1998). In addition, and in contrast to what was seen in the knee, a large increase in collagen type II synthesis and aggrecan turnover has been shown in human ankle joints with early lesions; the ankle joint showed an anabolic response with emphasis on repair, whereas the pathological development of the knee showed more of a catabolic response with collagen degradation (Aurich et al., 2005). Considering the results shown in our present study, it is intriguing that the ankle joint also seems more resistant to degeneration and add support to the consideration of rat joints as models of the human conditions. However, differences exist between rat and human cartilage. We found that the number of chondrocytes in the chondrons (chondrocytes surrounded by a narrow pericellular matrix) in both rat ankle and knee were composed of up to four cells, whereas in humans chondrons in the ankle are organized into clusters of two to four cells each, while chondrons in the knee are composed of single cells (Kuettner and Cole, 2005). Further work is warranted in order to advance our understanding of the underlying mechanisms making ankle joint chondrocytes resist catabolic factors, and find ways to stimulate rebuilding of the matrix.

This work was performed exclusively in male rats, and whether the results would be the same in female rats cannot be known. However, our study was done in young rats, and the OA risk assessment in humans does not find gender differences in young people - it́s not until later in life that women predominantly suffer from the condition.

In conclusion, the present work shows that ankle versus knee joint injection of MIA resulted in different behavioural profiles. Levels of biochemical mediators and histopathological analysis of the respective joints 28 days after MIA injection support the results. These differences may mirror what has been found in human patients with osteoarthritis, where the ankle is less frequently affected compared to the knee joint. Moreover the resistance of ankle chondrocytes against the catabolic effects of MIA injection should be investigated further and could give insights to new therapeutic approaches.

## Declaration of Competing Interest

The authors declare that they have no known competing financial interests or personal relationships that could have appeared to influence the work reported in this paper
